# Supplementary material for: Explainable ensemble learning method for OCT detection with transfer learning
Source: PLoS One. 2024 Mar 22;19(3):e0296175. doi: 10.1371/journal.pone.0296175 (PMC10959366; doi:10.1371/journal.pone.0296175)
Supplement: Attachment — Submitted filename: Reviewer_yjs_20230605.docx [file pone.0296175.s002.docx]

Reviewer#1

The authors have done good work on the title “Explainable ensemble learning method for OCT detection with transfer learning”. It will add new knowledge and new areas of research to the subject area compared with other published material.

1. It would be more appropriate for the authors to define abbreviations upon first appearance in the main text such as degeneration (AMD), diabetic macular edema (DME), Optical coherence tomography (OCT) and convolutional neural network (CNN).

Response: Thank you for your comment. We have defined the abbreviations that appear for the first time in the main text.

2. Figure 1 need more enhancement to be of good quality; image resizing.

Response: Thanks for pointing out this! We have improved the quality of figure 1.

3. It would be more appropriate for the authors to standardize the writing of the CNN networks’ names “Efficient_V2, and Resnet34” across the whole manuscript.

Response: Thank you for the suggestion!We have standardized the writing of the CNN network name "Efficientnet_v2, and Resnet34" throughout the manuscript.

4. The authors have inserted Equations 1,3, 4, 5 and 6 in the section of “The Ensemble models”, but it was not cited inside the manuscript. Kindly check it and perform the required amendment.

Response: Thanks a lot! We have checked and made amendement.

5. The authors have inserted Figures 3 and 4 in the section of “The Ensemble models”, but it was not cited inside the manuscript. Kindly check the guideline of the PLOS ONE journal and perform the required amendment.

Response: Thank you for your comment. We have made amendement. It is demonstrated as following:

“To more comprehensively compare the impact of pre-training on model performance, we used three different CNN networks, Alexnet, Efficientnet_v2, and Resnet34. Fig. 3 illustrates the performance comparison of three CNN networks between with and without pretrain in each class. One can see that the performance of all classes is significantly improved after loading pre-training parameters. In contrast, DME class has the best performance among them. The overall performance of all three networks is calculated and demonstrated in Tables 3 and 4. It is observed that the performance of all three networks was improved after loading pre-training parameters. Among them, the Resnet34 network loaded with pre-training has the strongest recognition ability, with an average AUC of 99.6%. The Efficientnet_v2 network has the most obvious improvement, with an AUC increase of 9.22% and a verification rate increase from 74.51% to 95.37%. After loading pre-training, the standard deviation (SD) of Alexnet, Efficientnet_v2, and Resnet34 models decreased by 3.45%, 2.97%, and 2.1%, respectively. Fig. 4 makes the accuracy comparison of three CNN models. It also demonstrates the Resnet34 network loaded with pre-training has the strongest recognition ability. This suggests that pre-training has improved the robustness of these models.”

6. In the section of “Analysis of the effectiveness of Ensemble models based on pre-training methods”, the authors stated that the ensemble model obtained the best classification performance with accuracy: 97.9±1.89. However, in Table 5, the authors represented the precision value not the accuracy value. Kindly check it and perform the required amendment.

Response: Thank you for your comment. It is a typo error. We rewrite the word “accuracy” to be “precision” in the updated manuscript.

7. In the section of “Analysis of the effectiveness of Ensemble models based on pre-training methods”, some of the calculations in the following sentences are not correct. Kindly check it and perform the required amendment.

“Efficientnet_v2 and Resnet34, and the F1 score has a significant improvement of 2.38, 2.55 and 1.15. Figure 5 shows the accuracy of the three CNNs models and the ensemble model. We observe that the ensemble model not only improves the accuracy, but also further improves the robustness of the model. Comparing Alexnet, Efficientnet_v2, and Resnet34, it improves the classification accuracy by 2.37%, 2.52%, and 1.15%,”.

Response: Thank you for the suggestion! We double check and rewrite it as following:

“The ensemble model compared Alexnet, Efficientnet_v2 and Resnet34, and the F1 score has a significant improvement of 2.38, 2.55 and 1.17. ”.

8. Authors should include the discussion section to interpret of their results and explain how the results relate to the hypothesis presented as the basis of the study and provide a succinct explanation of the implications of the findings, particularly in relation to previous related studies and potential future directions for research. I encourage the authors to present and discuss their findings concisely in discussion section.

Response: Thank you very much for pointing out the problem! We have discussion section to demonstrate our findings.

Reviewer#2

Moderate editing is required throughout the manuscript, for example:

1. In the abstract, “The accuracy and interpretability of artificial intelligence are fundamental to revolutionizing Optical Coherence Tomography (OCT) image detection, significantly reducing the grueling manual labor required of clinicians…….., with 15 samples in each category”. Moderate grammar editing is required.

Response: Thank you for the suggestion! We have rewritten these sentences as follows:

The accuracy and interpretability of artificial intelligence (AI) are crucial for the advancement of optical coherence tomography (OCT) image detection, as it can greatly reduce the manual labor required by clinicians. By prioritizing these aspects during development and application, we can make significant progress towards streamlining the clinical workflow. In this paper, we propose an explainable ensemble approach that utilizes transfer learning to detect fundus lesion diseases through OCT imaging. Our study utilized a publicly available OCT dataset consisting of normal subjects, patients with dry age-related macular degeneration (AMD), and patients with diabetic macular edema (DME), each with 15 samples.

2. “AMD) and DME are common a”. Moderate editing is required.

Response: Thank you for your comment. We have rewritten these sentences as follows:

Age-related macular degeneration (AMD) and diabetic macular edema (DME) are prevalent eye conditions that are becoming more common among elderly individuals and those with diabetes globally.

3. “For this study, the proposed algorithm was designed and evaluated on a publicly 138 available dataset[29].” Moderate editing is required.

Response: Thank you for your comment. We have rewritten these sentences as follows:

In this study, we designed and evaluated the proposed algorithm using a publicly available dataset [29].

4. “Specifically, the image preprocessing process in this paper is as follows. First, data enhancement is performed on the image by horizontal flip, vertical flip and a….). Moderate editing is required.

Response: Thanks a lot! We have rewritten these sentences as follows:

In this paper, the image preprocessing process involves data enhancement through horizontal and vertical flip as well as rotation within a range of -15° to 15°.

5. “Alexnet[30-32] is comprised of eight layers, including five convolutional layers and three fully connected layers”. Moderate editing is required.

Response: Thanks for pointing out this! We have rewritten these sentences as follows:

Alexnet [30-32] consists of a total of eight layers, which comprise of five convolutional layers and three fully connected layers.

6. “EfficientNetV2[34] is a family of image classification models, which offer superior parameter efficiency and faster…”. Moderate editing is required.

Response: Thank you for the suggestion!We have rewritten these sentences as follows:

Efficientnet_v2 [34] is a family of image classification models that provide improved parameter efficiency and faster training speeds compared to previous models.

7. “On the one hand, we trained the three frameworks from scratch. On the other hand, we loaded pre-trained model parameters to reduce the stress of training from scratch. The pre-training used in this paper was all performed on the ImageNet[35] database”. Moderate editing is required.

Response: Thank you for your comment.We have rewritten these sentences as follows:

To reduce the stress of training from scratch, we loaded the parameters of the pre-trained model trained from the ImageNet[35] database.

8. “The performances of our experiments are evaluated through Accuracy , Precision,”. Moderate editing is required.

Response: Thank you for your comment.We have rewritten these sentences as follows:

The performance of the experiments in this study was evaluated by accuracy, precision, recall, and F1 score.

9. “And the standard deviation (SD) of Alexnet, Efficient_v2 and Resnet34 models decreased by 3.45%, 254 2.97% and 2.1% after loading pre-training, which indicates the improved robustness 255 of these models”. Moderate editing is required.

Response: Thank you for your comment.We have rewritten these sentences as follows:

After loading pre-training, the standard deviation (SD) of Alexnet, Efficientnet_v2, and Resnet34 models decreased by 3.45%, 2.97%, and 2.1%, respectively. This suggests that pre-training has improved the robustness of these models.

10. “And the ensemble model has the most improvement inrecognizing DME, with an F1 score increase of 3.55%, 3.69% and 1.86% compared toAlexnet, Efficientnet_v2 and Resnet34”. Moderate editing is required.

Response: Thank you for your comment.We have rewritten these sentences as follows:

The ensemble model demonstrated the greatest improvement in recognizing DME, achieving an increase in F1 score of 3.55%, 3.69%, and 1.86% compared to Alexnet, Efficientnet_v2, and Resnet34, respectively.
