# Supplementary material for: Explainable ensemble learning method for OCT detection with transfer learning
Source: PLoS One. 2024 Mar 22;19(3):e0296175. doi: 10.1371/journal.pone.0296175 (PMC10959366; doi:10.1371/journal.pone.0296175)
Supplement: Attachment — Submitted filename: Response to Reviews.docx [file pone.0296175.s004.docx]

Dear Editor, Dear reviewers,

Thanks very much for taking your time to review this manu. I really appreciate all your comments and suggestions! Please find my itemized responses in below and my revisions/corrections in the re-submitted files.

**Reviewer#3**

(1) For Figure 6, please add the color code to the maps. Also, please add discussion about “why” as well as if this can be further enhanced using other explainable methods, e.g., LIME.

Response: Thank you for expert advice! In the updated manuscript, we make the following supplements in the discussion:

“Grad-CAM generates heatmap-like results, highlighting the image areas where the model activates a specific category, offering insights into spatial locations. However, Grad-CAM results can sometimes appear coarse, complicating the identification of precise features guiding the model's decisions. In contrast, LIME specializes in interpreting individual predictions, offering explanations that pinpoint pixel regions influencing each prediction. The two approaches complement each other: LIME delivers detailed, granular explanations, while Grad-CAM provides intuitive spatial information. Together, they may create a more comprehensive understanding of the model's decision-making process.”

(2) The discussion section lacks many aspects, such as limitation, how the methods can be applied to clinical settings, what is the future work, etc.

Response: Thanks for pointing out this! According to this instruction, we make the following supplements in the discussion:

“Our proposed approach exhibits promising results in the detection of retinal OCT images. However, it is crucial to acknowledge certain limitations. In this study, our primary focus was on detecting fundus diseases, specifically dry AMD and DME. Nonetheless, it is essential to consider the various other retinal diseases that may emerge within a clinical setting. The performance of the model and its applicability to other diseases still remain uncertain.

In our future work, we intend to enhance the dataset in order to improve generalization and encompass a broader spectrum of retinal diseases. Additionally, our aim is to perform practical validation in a clinical setting by seamlessly integrating artificial intelligence models into existing clinical workflows and effectively utilizing them. This will facilitate the incorporation of deep learning models into routine clinical practice.”

(3) I am not sure why the authors add refs [43] and [44] to the conclusions section. Please make future direction as general to specific problem, not a specific approach, and explain more (in the discussion) what modifications should be done, challenges, etc.

Response: Thanks for pointing out this! According to this instruction, we make the following supplements in the discussion:

“In exploring the future trajectory for ensemble CNN models, it is evident that their potential extends across various domains and problem landscapes. One promising avenue for advancement lies in tailoring ensemble architectures to specific problem rather than pursuing a one-size-fits-all domains approach . This necessitates a shift towards domain-centric modifications wherein ensemble structures are customized to capture nuanced features inherent to the problem at hand. Moreover, challenges persist in enhancing the diversity and robustness of ensemble constituents while ensuring computational efficiency. To address these challenges, future Endeavors could focus on designing novel strategies to enhance diversity among individual models within ensembles and optimize their integration for improved predictive performance.”

(4) Also, evaluation on a single data set is not suggested. The authors need to verify the robustness of their methods in additional data sets. There are publicly available OCTs data sets (e.g., UCSD dataset). Comparison with the peer methods that utilized the same data set should be included.

Response: Thanks for pointing out this! In the updated manuscript, we compare our method with other existing methods. At first, we compare the proposed method with Alexnet [38]，Efficientnet [34], Resnet34 [41] CNN models. Furthermore, we compare it with the other machine learning models . All demonstrate that the proposed ensemble methods has best performance. we make the following supplements in the introduction:

“In addition to comparing with existing Alexnet, Efficientnet_v2, and Resnet34 CNN models, we further compared it with other existing methods. In [47], Wang et al. used N-Gram-Based Model for OCT classification, and the optimal accuracy obtained was 93.3%, and the AUC was 99.85%. In [48], the authors applied the Surrogate-Assisted CNN model for OCT classification, and obtained an accuracy of 95.09% and an AUC value of 98.56%. The optimal accuracy of the algorithm recommended in this article is greater than 98%, and the AUC is greater than 98.8%. It is significantly better than the above two models. This fully demonstrates the effectiveness of the proposed ensemble learning method.”

(5) The authors need to separate the introduction from the related work. Additionally, the literature work lacks some approaches that employed ensemble/computational learning for OCT classification e.g., Huang et al. Front. Neurosci. 2023; Ai et al. Front. Neuroinform. 2022, Akinniyi et al., Bioengineering 2023; Kayadibi et al. 2023 Int. J. Comput. Intell. Syst.

Response: Thank you for your comment. According to the good instruction, we make the following supplements in the introduction:

Huang et al. [33] introduced a novel global attention block (GAB) that enhances classification performance when integrated with any CNN, resulting in a notable improvement of 3.7% accuracy compared with the EfficientNetV2B3 model. Ai et al. [34] proposed FN-OCT, a fusion network-based algorithm for retinal OCT classification that fuses predictions from InceptionV3, Inception-ResNet, and Xception classifiers with CBAM, employing three fusion strategies. On the dataset from UCSD, FN-OCT achieved 98.7% accuracy and 99.1% AUC, surpassing InceptionV3 by 5.3%. Akinniyi et al. [35] proposed a multi-stage classification network using OCT images for retinal image classification. Their architecture utilizes a pyramidal feature ensemble built on DenseNet for extracting multi-scale features. Evaluation on two datasets demonstrates advantages, achieving high accuracies for binary, three-class, and four-class classification. Kayadibi et al. [36] proposed a hybrid approach for retinal disease identification. It uses a fully dense fusion neural network (FD-CNN) and dual preprocessing to reduce speckle noise, extract features, and generate heat maps for diagnosis confidence.

(6) Table 4, 5, and 6 need statistical analysis of the reported values and Figures 4&5 are not necessary to as Tables are sufficient.

Response: Thank you for underlining this deficiency. Figure 4&5 illustrates Accuracy performance of three CNNs models and ensemble model, and Table 4, 5, and 6 illustrates Precision, Recall, F1_score and AUC performances. Accuracy and F1 score are both indicators used to evaluate the performance of classification models, but they focus on slightly different aspects. They are used together to provide a comprehensive evaluation of model performance. In Table 4, 5, and 6, we already did statical analysis including mean value and variance to demonstrate the effectiveness of the proposed ensemble model.

(7) Please review the manuscript writing and language. For example, instead of “L. R. Ashok et al.” it should be only “Ashok et al.”

Response: Thanks a lot! We revised the manuscript in accordance with the reviewers' comments and carefully proof-read the manuscript.

(8) The abbreviation should be doen one time, e.g., age-related macular degeneration (AMD) is used in page 3 and 4.

Response: Thanks a lot! We have made correction according to the comments.
