# Supplementary material for: Explainable ensemble learning method for OCT detection with transfer learning
Source: PLoS One. 2024 Mar 22;19(3):e0296175. doi: 10.1371/journal.pone.0296175 (PMC10959366; doi:10.1371/journal.pone.0296175)
Supplement: Attachment — Submitted filename: reviewer comments - Mai-25032023 - Copy.docx [file pone.0296175.s001.docx]

**Reviewer Comments**

Dear Editor(s),	March 21, 2023

I am pleased to submit my comments for the submitted manuscript (**PONE-D-23-05848)** entitled “**Explainable ensemble learning method for OCT detection with transfer learning”** to be taken into consideration for publication by your esteemed journal “***PLOS ONE***”.

Age-related macular degeneration (AMD), and diabetic macular edema (DME) are common and increasingly prevalent eye conditions among elderly individuals and those with diabetes worldwide. As the leading causes of irreversible vision loss worldwide, AMD and DME require reliable and accurate diagnostic and monitoring tools. The optical coherence tomography (OCT) has become a critical imaging technique for diagnosing and tracking patients with AMD and DME, offering a non-invasive and touch-free solution. Its application could help healthcare professionals detect these conditions early and initiate prompt and effective treatment plans, potentially improving patient outcomes and quality of life. The accuracy and interpretability of artificial intelligence are fundamental to OCT image detection, significantly reducing the gruelling manual labour required of clinicians. Prioritizing these aspects in development and application will help in taking significant strides toward streamlining the clinical workflow.

Therefore, the aim of this study is to develop of an explainable transfer ensemble model to effectively identify age-related fundus macular degeneration, which is an ensemble architecture based on migration learning, and to illustrate the learning content of the model through Class activation mapping (CAM) as well as gradient - weighted Class Activation Mapping (Grad-CAM) visualization .The proposed ensemble architecture will help the model to reduce the variance in predictions compared to a single independent model.

In this study, the authors have successfully developed an explainable ensemble approach with transfer learning in detecting fundus lesion diseases using OCT imaging. A publicly available OCT dataset including normal subjects, patients with dry AMD, and 21 patients with DME were used, with 15 samples in each category. The impact of pre-trained weights on the performance of individual networks was first compared, and then these networks were ensemble using majority soft polling. Finally, the features learned by the networks were visualized using Grad-CAM and CAM. The use of pre-trained ImageNet weights improved the performance from 68.17% to 92.89%. The ensemble model consisting of the three CNN models with pre-trained parameters loaded performed best, correctly distinguishing between AMD patients, DME patients and normal subjects 100% of the time. Visualization results showed that Grad-CAM could display the lesion area more accurately. It is demonstrated that the proposed approach could have good performance of both accuracy and interpretability in retinal OCT image detection.

The authors have done good work on the title “Explainable ensemble learning method for OCT detection with transfer learning”. It will add new knowledge and new areas of research to the subject area compared with other published material.

**However, i have some minor concerns:**

1. It would be more appropriate for the authors to define abbreviations upon first appearance in the main text such as degeneration (AMD), diabetic macular edema (DME), Optical coherence tomography (OCT) and convolutional neural network (CNN).
2. Figure 1 need more enhancement to be of good quality; image resizing.
3. It would be more appropriate for the authors to standardize the writing of the CNN networks’ names “Efficient_V2, and Resnet34” across the whole manuscript.
4. The authors have inserted Equations 1,3, 4, 5 and 6 in the section of “The Ensemble models”, but it was not cited inside the manuscript. Kindly check it and perform the required amendment.
5. The authors have inserted Figures 3 and 4 in the section of “The Ensemble models”, but it was not cited inside the manuscript. Kindly check the guideline of the PLOS ONE journal and perform the required amendment.
6. In the section of “Analysis of the effectiveness of Ensemble models based on pre-training methods”, the authors stated that the ensemble model obtained the best classification performance with accuracy: 97.9±1.89. However, in Table 5, the authors represented the precision value not the accuracy value. Kindly check it and perform the required amendment.
7. In the section of “Analysis of the effectiveness of Ensemble models based on pre-training methods”, some of the calculations in the following sentences are not correct. Kindly check it and perform the required amendment.

“Efficientnet_v2 and Resnet34, and the F1 score has a significant improvement of 2.38, 2.55 and 1.15. Figure 5 shows the accuracy of the three CNNs models and the ensemble model. We observe that the ensemble model not only improves the accuracy, but also further improves the robustness of the model. Comparing Alexnet, Efficientnet_v2, and Resnet34, it improves the classification accuracy by 2.37%, 2.52%, and 1.15%,”.

8. Authors should include the discussion section to interpret of their results and explain how the results relate to the hypothesis presented as the basis of the study and provide a succinct explanation of the implications of the findings, particularly in relation to previous related studies and potential future directions for research. I encourage the authors to present and discuss their findings concisely in discussion section.
9. **Moderate editing is required throughout the manuscript, for example:**
1. In the abstract, “The accuracy and interpretability of artificial intelligence are fundamental to revolutionizing Optical Coherence Tomography (OCT) image detection, significantly reducing the grueling manual labor required of clinicians…….., with 15 samples in each category”. Moderate grammar editing is required.
2. “AMD) and DME are common a”. Moderate editing is required.
3. “For this study, the proposed algorithm was designed and evaluated on a publicly 138 available dataset[29].” Moderate editing is required.
4. “Specifically, the image preprocessing process in this paper is as follows. First, data enhancement is performed on the image by horizontal flip, vertical flip and a….). Moderate editing is required.
5. “Alexnet[30-32] is comprised of eight layers, including five convolutional layers and three fully connected layers”. Moderate editing is required.
6. “EfficientNetV2[34] is a family of image classification models, which offer superior parameter efficiency and faster…”. Moderate editing is required.
7. “On the one hand, we trained the three frameworks from scratch. On the other hand, we loaded pre-trained model parameters to reduce the stress of training from scratch. The pre-training used in this paper was all performed on the ImageNet[35] database”. Moderate editing is required.
8. “The performances of our experiments are evaluated through Accuracy , Precision,”. Moderate editing is required.
9. “And the standard deviation (SD) of Alexnet, Efficient_v2 and Resnet34 models decreased by 3.45%, 254 2.97% and 2.1% after loading pre-training, which indicates the improved robustness 255 of these models”. Moderate editing is required.
10. “And the ensemble model has the most improvement inrecognizing DME, with an F1 score increase of 3.55%, 3.69% and 1.86% compared toAlexnet, Efficientnet_v2 and Resnet34”. Moderate editing is required.

Best regards,

Dr. Mai Abdel Haleem Abusalah

Faculty of Medical Allied Science,

Zarqa University,

Zarqa, 13110, Jordan.

Tel: +962-796862347

e-mail: [ellamomo88@yahoo.com](mailto:ellamomo88@yahoo.com)
