# Supplementary material for: Explainable ensemble learning method for OCT detection with transfer learning
Source: PLoS One. 2024 Mar 22;19(3):e0296175. doi: 10.1371/journal.pone.0296175 (PMC10959366; doi:10.1371/journal.pone.0296175)
Supplement: Attachment — Submitted filename: reviewer comments - Mai-01072023.docx [file pone.0296175.s003.docx]

**Reviewer Comments**

**Dear Editor(s), July 01, 2023**

I am pleased to submit my comments for the submitted manuscript (**PONE-D-23-05848R1**) entitled “**Explainable ensemble learning method for OCT detection with transfer learning**” to be taken into consideration for publication by your esteemed journal “***PLOS ONE***”.

Age-related macular degeneration (AMD), and diabetic macular edema (DME) are common and increasingly prevalent eye conditions among elderly individuals and those with diabetes worldwide. As the leading causes of irreversible vision loss worldwide, AMD and DME require reliable and accurate diagnostic and monitoring tools. The optical coherence tomography (OCT) has become a critical imaging technique for diagnosing and tracking patients with AMD and DME, offering a non-invasive and touch-free solution. Its application could help healthcare professionals detect these conditions early and initiate prompt and effective treatment plans, potentially improving patient outcomes and quality of life. The accuracy and interpretability of artificial intelligence are fundamental to OCT image detection, significantly reducing the gruelling manual labour required of clinicians. Prioritizing these aspects in development and application will help in taking significant strides toward streamlining the clinical workflow.

Therefore, the aim of this study is to develop of an explainable transfer ensemble model to effectively identify age-related fundus macular degeneration, which is an ensemble architecture based on migration learning, and to illustrate the learning content of the model through Class activation mapping (CAM) as well as gradient-weighted Class Activation Mapping (Grad-CAM) visualization. The proposed ensemble architecture will help the model to reduce the variance in predictions compared to a single independent model.

In this study, the authors have successfully developed an explainable ensemble approach with transfer learning in detecting fundus lesion diseases using OCT imaging. A publicly available OCT dataset including normal subjects, patients with dry AMD, and 21 patients with DME were used, with 15 samples in each category. The impact of pre-trained weights on the performance of individual networks was first compared, and then these networks were ensemble using majority soft polling. Finally, the features learned by the networks were visualized using Grad-CAM and CAM. The use of pre-trained ImageNet weights improved the performance from 68.17% to 92.89%. The ensemble model consisting of the three CNN models with pre-trained parameters loaded performed best, correctly distinguishing between AMD patients, DME patients and normal subjects 100% of the time. Visualization results showed that Grad-CAM could display the lesion area more accurately. It is demonstrated that the proposed approach could have good performance of both accuracy and interpretability in retinal OCT image detection.

The authors have done good work on the title “**Explainable ensemble learning method for OCT detection with transfer learning**”. It will add new knowledge and new areas of research to the subject area compared with other published material.

The authors have adequately addressed all comments and performed the required amendments; hence I highly recommend accepting this interesting article.

Best regards,

Dr. Mai Abdel Haleem Abusalah

Faculty of Medical Allied Science,

Zarqa University,

Zarqa, 13110, Jordan.

Tel: +962-796862347

e-mail: [ellamomo88@yahoo.com](mailto:ellamomo88@yahoo.com)

Best regards,
